# Advantages and Pitfalls of Capillary Electrophoresis of Pharmaceutical Compounds and Their Enantiomers in Complex Samples: Comparison of Hydrodynamically Opened and Closed Systems

**DOI:** 10.3390/ijms21186852

**Published:** 2020-09-18

**Authors:** Marián Masár, Jasna Hradski, Martin G. Schmid, Roman Szucs

**Affiliations:** 1 Department of Analytical Chemistry, Faculty of Natural Sciences, Comenius University in Bratislava, Mlynská Dolina CH2, Ilkovičova 6, SK-84215 Bratislava, Slovakia; marian.masar@uniba.sk (M.M.); hradski1@uniba.sk (J.H.); 2 Department of Pharmaceutical Chemistry, Institute of Pharmaceutical Sciences, University of Graz, Universitätsplatz 1, A-8010 Graz, Austria; martin.schmid@uni-graz.at; 3 Pfizer R & D UK Limited, Ramsgate Road, Sandwich CT13 9NJ, UK

**Keywords:** Capillary electrophoresis, hydrodynamically opened and closed systems, chiral separation, pharmaceutical analysis, serum analysis

## Abstract

Several research disciplines require fast, reliable and highly automated determination of pharmaceutically active compounds and their enantiomers in complex biological matrices. To address some of the challenges of Capillary Electrophoresis (CE), such as low concentration sensitivity and performance degradation linked to the adsorption and interference of matrix components, CE in a hydrodynamically closed system was evaluated using the model compounds Pindolol and Propranolol. Some established validation parameters such as repeatability of injection efficiency, resolution and sensitivity were used to assess its performance, and it was found to be broadly identical to that of hydrodynamically opened systems. While some reduction in separation efficiency was observed, this was mainly due to dispersion caused by injection and it had no impact on the ability to resolve enantiomers of model compounds even when spiked into complex biological matrix such as blood serum. An approximately 18- to 23-fold increase in concentration sensitivity due to the employment of wide bore capillaries was observed. This brings the sensitivity of CE to a level similar to that of liquid chromatography techniques. In addition to this benefit and unlike in hydrodynamically opened systems, suppression of electroosmotic flow, which is essential for hydrodynamically closed systems practically eliminates the matrix effects that are linked to protein adsorption.

## 1. Introduction

The determination of Active Pharmaceutical Ingredients (APIs) in biological fluids such as serum, plasma or urine is essential for several research disciplines such as drug pharmacology, drug metabolism, pharmacokinetics, as well as forensic and clinical toxicology. Routine, high throughput quantitative determinations of these compounds require that applied analytical techniques are sufficiently simple to perform while amenable to automation as well as providing adequate selectivity and sensitivity [1]. Capillary Electrophoresis (CE) is particularly suitable for the analysis of diverse types of APIs covering cationic, anionic as well as neutral analytes as it offers a wide range of separation modes in combination with a wide variety of detection techniques. Short analysis times, high resolution and high separation efficiency, in addition to low reagent costs and minimal sample requirements, make this technique particularly attractive [2,3]. Besides the obvious advantages, the analysis of APIs in complex biological samples can often be challenging due to multiple interferences from matrix components. An example of these are proteins, which are often present in these samples at very high concentrations and have a tendency to adsorb onto the inner wall of bare fused silica capillaries. This can lead to unpredictable fluctuations of electroosmotic flow (EOF) and consequently can cause highly variable migration velocities of analytes [4]. Slow rates of association and dissociation processes involved in the interaction of some of the analytes with proteins can significantly impair separation efficiencies due to electrodiffusion [5,6]. Additionally, biological fluids usually contain high concentrations of salts, which can have an adverse effect on the resolution of some analytes due to an increase in the electromigration dispersion of the matrix components. Besides high salt content, biological fluids also represent relatively complex systems with multiple chemical components present. This can lead to either an increased risk of comigrations with the analytes of interest especially when non-selective detection techniques are employed or signal suppression when mass selective detection is used. Due to low therapeutic concentrations of most pharmaceutical drugs their concentration levels in biological fluids are very low (ppb to ppm) [7]. Detection sensitivity of CE is, however, generally considered inferior to other separation techniques such as High-Performance Liquid Chromatography (HPLC). This is due to the restriction on sample injection volume (nL) because of the small internal diameter (i.d.) of the capillary tubes employed as separation compartments.

The vast majority of CE applications is carried out using capillaries with i.d. between 20–75 µm. This restriction is imposed due to the hydrodynamic flow dispersion [8], which becomes the prevalent cause of dispersion in capillaries of larger i.d. [9]. Additionally, larger i.d. capillaries are prone to excessive heat generation associated with the passage of electric current through them. This also contributes to zone dispersion, but in extreme cases, it can cause thermal degradation of analytes or breakdown of the current due to buffer boiling [10]. The moderate concentration sensitivity of current CE detection systems constitutes a significant challenge in achieving the required limits of detection (LOD) or quantitation for contemporary industrial applications. To overcome some of these challenges, extensive sample pre-treatment has become common practice in the analysis of biological fluids. This is carried out with two major objectives: (1) the removal of potentially interfering matrix components (sample clean-up); and (2) an increase of analyte concentration (sample pre- concentration).

An alternative way of increasing sample load-ability is to carry out CE analysis in wide bore (320 µm i.d.) capillary tubes. This wide bore capillary format also provides a longer optical path when compared to the conventionally used 50–75 µm i.d. capillaries thus improving detection sensitivity. To overcome the above-mentioned challenges posed by hydrodynamic dispersion, CE experiments employing wide bore capillaries can be performed in an hydrodynamically closed separation system (CSS). Such a system, originally developed for Capillary Isotachophoresis, but also applied in CE [8,11,12], prevents any hydrodynamically induced movement of the separation medium. In fact, under hydrodynamically closed conditions, any bulk movement of a separation medium must be prevented [11]. Papers dealing with various tools for effective and reproducible suppression of the EOF in CE capillaries can be found elsewhere [4,13,14,15].

In this contribution, we carried out a feasibility study aimed at investigating the performance of CE as applied to the analysis of APIs and their enantiomers in serum using 50 and 320 µm i.d. capillary tubes. The potential benefits of wide bore tubes were assessed against the expected increase of thermal dispersion due to inherent thermal effects. The electroosmotic dispersion typically associated with operations in a closed environment was eliminated by suppression of EOF through dynamic coating of the capillary walls. The impact of this surface modification on the reduction of the serum protein adsorption on capillary walls, which in turn leads to the improvements of the reproducibility of the electrokinetic velocities of analytes was also studied.

## 2. Results and Discussion

### 2.1. Comparison of Hydrodynamically Opened and Closed Separation Systems

An hydrodynamically opened separation system (OSS) is the most common experimental setup for performing CE experiments in which reservoirs containing separation medium are exposed to atmospheric pressure. The capillary, which is filled with the separation medium (buffer), is fixed between these reservoirs. In this most basic experimental setup, analytes migrate from one end of the capillary (injection end) to the other end of the capillary (detection end) by a combination of electroosmotic movement and electrophoretic migration. The latter is facilitated by the analyte charge and electric field applied across the capillary. Separation of analyte zones is achieved by differences in their intrinsic electrophoretic mobilities (µ_ep_).

CE in OSS can be performed either with or without EOF. EOF is a flow of bulk liquid which originates from charge separation between the capillary wall and the separation medium. Its direction depends on the charge of the excess ions in the solution. If the electric field across the capillary is maintained constant by keeping the concentration of analytes low relative to the concentration of the separation buffer, then the EOF does not contribute to the dispersion of separated zones [8,16] due to its plug-like velocity profile. Under these circumstances EOF affects ion mobilities of all analytes equally, as it reduces or increases the magnitude of their electrophoretic velocity. Although EOF has a great influence on resolution in CE, many applications require its elimination in order to prevent electrostatic adsorption of analytes on capillary walls. EOF elimination can be achieved by means of either static chemical modification or dynamic capillary wall modification [4]. The latter approach is more flexible, and requires the addition of EOF suppressors to the separation medium [11,17]. Hydrodynamic flow (HDF) across a capillary, which can be generated by hydrostatic pressure difference between both capillary ends, is generally detrimental to the performance of CE as it adds to the dispersion of separated zones due to its parabolic velocity profile [18]. This flow is eliminated by ensuring that the level of fluid in both reservoirs is equal throughout the separation process and by limiting the i.d. of capillaries typically to 75 µm.

In hydrodynamically closed separation systems (CSS), the detection end of the capillary is usually separated from the buffer reservoir by a mechanical barrier in the form of a semipermeable membrane [18,19]. This means that one end of the capillary is practically blocked for the bulk of the separation medium as only ions can be electrophoretically transported across this membrane. This prevents the build-up of the hydrostatic pressure difference and eliminates HDF [18]. With CSS it is, therefore, possible to use capillaries with a larger i.d. As the zeta-potential of the membrane differs from that of the separation compartment, this gives rise to volume velocity differences and consequently increases the dispersion of separated zones through so called electroosmotic dispersion [18]. It is, therefore, necessary to suppress EOF when performing CE separations in CSS.

**Figure 1:**
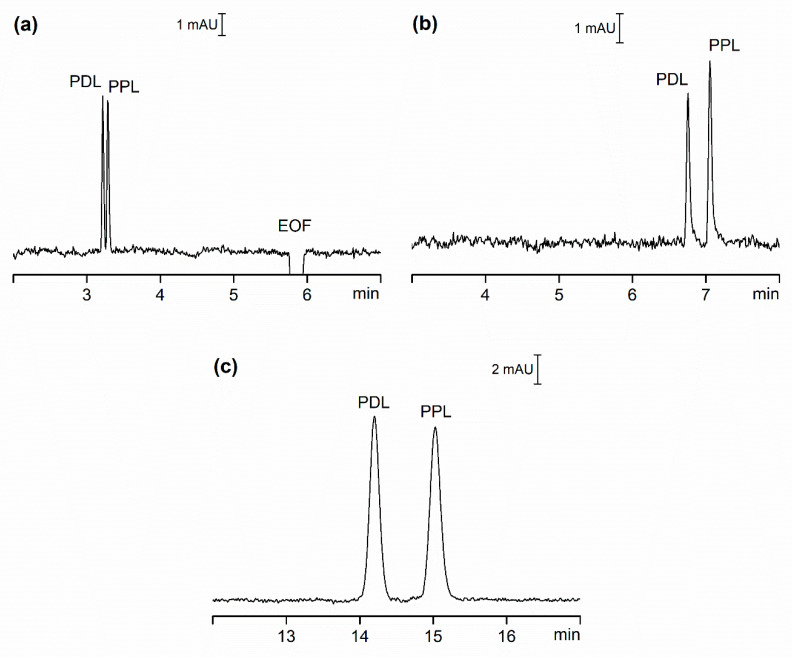
CE separation of PDL and PPL, each prepared at concentration of 5 µmol/L (CSS) and 20 µmol/L (OSS-EOF and OSS-sEOF). (**a**) OSS-EOF Buffer: 30 mmol/L β-alanine, 10 mmol/L poly(ethylene glycol) dicarboxylic acid, 0.1 mmol/L DETA; pH = 3.7; conditions: 25 kV (approximately 3.7 µA), 21 °C, detection at 210 nm. (**b**) OSS-sEOF Buffer: as (**a**) with addition of 0.05% MHEC; conditions: as (**a**). (**c**) CSS Buffer: as (**b**); conditions: as (**a**) except 75 µA (approximately 2.80 kV). See Materials and Methods for all other details.

To compare OSS and CSS, we carried out CE analysis of two model pharmaceutical compounds, Pindolol (PDL) and Propranolol (PPL). Estimated basic pKa values of these compounds is approximately 9.5, calculated using ACD Percepta software (ACD/Labs, Toronto, ON, Canada), and at the pH of the separation buffer (pH = 3.7) they possess a positive charge. Figure 1 shows the separation of these two compounds under three scenarios: a) in OSS with EOF (OSS-EOF), b) in OSS with suppressed EOF (OSS-sEOF), and c) in CSS with suppressed EOF (CSS). The separation buffer for all systems was identical except for systems with suppressed EOF, where 0.05% (*w/v*) methylhydroxyethylcelullose (MHEC) was added. 0.1 mmol/L of diethylenetriamine (DETA) was also added to the separation buffer to prevent compound adsorption on the walls of separation compartments [20].

In our attempt to compare the three systems, we focused on four criteria: repeatability of migration times, separation efficiency, resolution and sensitivity. To eliminate the impact of instrument set-up as much as possible we selected detectors with nearly identical setting for both OSS and CSS experiments. Table 1 lists some of the data for these three systems expressed as the average of ten repeated injections of the model sample.

#### 2.1.1. Repeatability

As stated in the Introduction, in CE the movement of compounds results from a combination of electroosmotic and electrophoretic velocity. If present, HDF will also affect the ion movement. Consequently, total variability of migration time also consists of contributions from EOF, HDF and electrophoretic movement [18]. In line with data in Table 1 we observed that the repeatability of migration times in the three systems under consideration is broadly identical. This observation is further reinforced by the broadly identical repeatability of peak areas as in CE the observed peak areas are affected by the velocity of migration of the separated zones [22,23].

#### 2.1.2. Efficiency and Resolution

Although, due to the plug-like profile of EOF, its contribution to zone dispersion should be negligible, from the data in Table 1 we observed an approximately 50% (PDL) to 55% (PPL) increase of separation efficiency (N) for the hydrodynamically opened system with suppressed EOF (OSS-sEOF) when compared to a system with EOF present (OSS-EOF). Apart from the addition of the EOF suppressor to the separation buffer in the form of 0.05% MHEC, all other experimental conditions were kept the same. This increase in efficiency can only in part be ascribed to extra dispersion due to the EOF, and the more likely explanation is that the EOF suppressor reduces adsorption of positively charged components on capillary walls as previously described [14,17]. The increase of separation efficiency is also accompanied by the increase of resolution between PDL to PPL from 1.47 (OSS-EOF) to 3.64 (OSS-sEOF). An approximately 65% (PDL) to 70% (PPL) reduction in efficiency when compared to OSS-sEOF was observed when analyzing the model sample using the CSS as shown in Table 1. While the sample and the separation buffer were identical, some other experimental conditions, such as applied current, internal and external diameters as well as the length of the separation capillary and injection plug length, were different in CSS, potentially giving rise to the increase in extra column band broadening. The total difference in plate height between OSS-sEOF and CSS was calculated as

(1) $${\Delta H}_{\mathrm{diff},\mathrm{tot}}{\;=\;H}_{\mathrm{CSS}}{-H}_{\mathrm{OSS}-\mathrm{sEOF}}=\frac{L_{d,\;\mathrm{CSS}}}{N_{\mathrm{CSS}}}-\frac{L_{d,\;\mathrm{OSS}-\mathrm{sEOF}}}{N_{\mathrm{OSS}-\mathrm{sEOF}}}$$

where the L_d,CSS_ and L_d,OSS-sEOF_ correspond to the distance from the point of injection to the detector for the CSS and OSS-sEOF, respectively. Then ΔH_diff,tot_ is 4.02 µm for PDL and 4.73 µm for PPL (See Table 1 for N_CSS_ and N_OSS-sEOF_). To assess the injection contribution to the dispersion, the injection plug length had to be estimated for CSS and OSS-sEOF. For CSS, the injected volume (V_inj_) is fixed at approximately 200 nL, and the sample plug length L_inj_ = 3.2 mm was measured by injecting a solution of dye. OSS employs hydrodynamic injection and the injected volume is calculated according to the Poiseuille-Hagen relationship

(2) $$V_{\mathrm{inj}}=\;\frac{\Delta {\mathrm{Ptd}}^{4}\pi }{128L\eta }$$

where ΔP = 2000 Pa is applied injection pressure, t = 6 s is injection time, L = 70 cm is capillary length and η = 8.9 × 10^−4^ Pas is the sample viscosity.

Injection plug length can then be calculated (d = capillary diameter)

(3) $$L_{\mathrm{inj}}{\;=\;V}_{\mathrm{inj}}/\left(\pi {\frac{d}{4}}^{2}\right)\;=\;1.5\;\mathrm{mm}$$

The difference in plate height originating from the injection and contributing to the total difference in plate height was calculated as

(4) $${\Delta H}_{\mathrm{diff},\;\mathrm{inj}}{\;=\;H}_{\mathrm{inj},\;\mathrm{CSS}}{-H}_{\mathrm{inj},\;\mathrm{OSS}-\mathrm{sEOF}\;}=\;\frac{L_{\mathrm{inj},\;\mathrm{CSS}}^{2}}{{12L}_{d,\;\mathrm{CSS}}}-\frac{L_{\mathrm{inj},\;\mathrm{OSS}-\mathrm{sEOF}}^{2}}{{12L}_{d,\;\mathrm{OSS}-\mathrm{sEOF}}}\;=\;3.56\;\mu m\;$$

As the detector slit size was identical (6 nm) in OSS-sEOF and CSS, the detector contribution to the total dispersion was negligible and it was ignored, as it was several orders of magnitude smaller than the contribution from the injection. The latter accounts for an 89% (PDL) to 75% (PPL) increase of the total plate height in CSS. This means that the contribution from diffusion and thermal effects only account for the remaining 12–25% of the total plate height difference. This proportion is perhaps unexpectedly small; however, one must take into account that only a modest increase in power was generated in CSS when compared to OSS-sEOF (0.09 W for OSS-sEOF vs. 0.21 W for CSS).

Despite a significant reduction in efficiency, the resolution between PDL and PPL was only marginally reduced from 3.64 (OSS-sEOF) to 3.23 (CSS).

#### 2.1.3. Sensitivity

One of the reasons why the full potential of CE has not been realized, especially in the field of pharmaceutical analysis, is its inferior concentration sensitivity when compared to other separation techniques. This is mainly due to the above-mentioned restrictions on i.d. of capillaries when they are employed as separation compartments. It not only reduces the detector light-path, but it also limits the injection volume. While the concentration sensitivity expressed as LOD (µmol/L) remained broadly identical for OSS-EOF and OSS-sEOF, an approximate 18- to 23-fold improvement in concentration sensitivity was observed for CSS (Table 1). This is mainly due to a 6.4-fold increase of the optical path length. The wider capillary i.d. also permits a larger injection volume (257 nL in CSS vs. 2.9 nL in OSS) while maintaining the narrow injection sample plug length.

If the typical reporting limit for impurities in API is 0.05% (*w/w*) [24] then assuming the nominal main component concentration of 0.1 mg/mL the required limit of quantitation (LOQ) should be 0.05% [25], and this corresponds to an LOD approximately at the 0.02% level. As can be seen in Table 2, this is achievable for CSS; however, for OSS, the achieved sensitivity is over an order of magnitude worse than required.

This brings the concentration sensitivity of CE in CSS setup close to that of a modern Ultra High-Performance Liquid Chromatography (UHPLC). Similarly, for the absolute injected amount, if for the UHPLC we assume a typical sample concentration of 0.1 mg/mL and an injection volume of 2 µL, then for the LOD at 0.02% (*w/w*) of the nominal loading of the main component, we inject approximately 40 pg of each component. For CSS, the absolute amount of injected PDL and PPL at LOD level is 4 and 5 pg, respectively, which demonstrates the superior mass sensitivity of this technique.

### 2.2. Comparison of Hydrodynamically Opened and Closed Separation Systems for Chiral Separations

CE is often considered a technique of choice for chiral separations [26,27,28,29]. This is mainly due to its simplicity, speed of chiral screening and low cost when compared to other separation techniques such as chiral HPLC. In chiral HPLC method development, several chiral columns are usually screened either sequentially [30,31] or in parallel [32,33,34] in combination with multiple mobile phases. Although ultimately successful, this approach is costly due to the significant cost of chiral stationary phases. It is also time consuming and generates a significant amount of waste. In CE, on the other hand, a small amount of chiral selector is usually added to the separation buffer. Chiral selectors can be at the pH of the separation buffer, either neutral or charged. If they are neutral, then they either move with EOF or, in the absence of EOF, are stationary. When charged they can move in the same or opposite direction to analytes. The enantioselectivity is achieved by differential interaction of enantiomers with the chiral selector.

**Figure 2:**
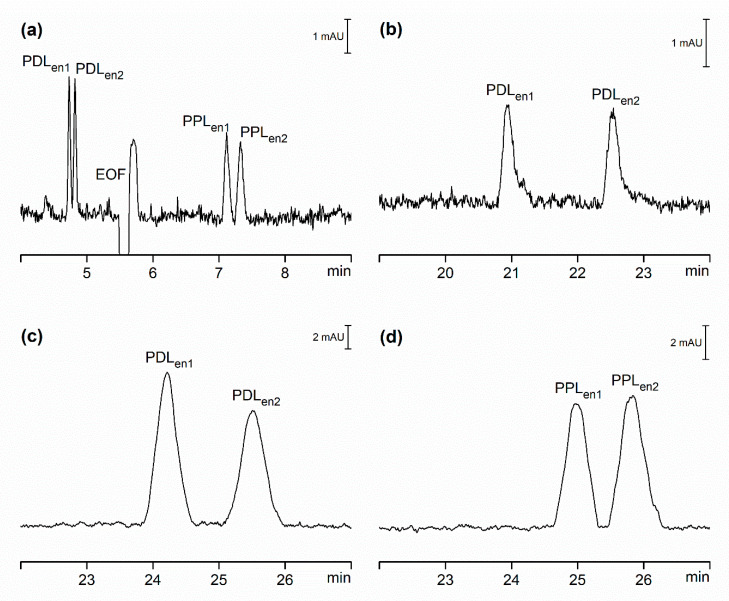
CE separation of PDL and PPL enantiomers, each prepared at concentration of 5 µmol/L (CSS) and 20 µmol/L (OSS-EOF and OSS-sEOF). (**a**) OSS-EOF Buffer: 30 mmol/l β-alanine, 5 mmol/L poly(ethylene glycol) dicarboxylic acid, 0.1 mmol/L DETA, 2 mmol/L CMBCD; pH = 3.8; conditions: 25 kV (approximately 4.5 µA), 21 °C, detection at 210 nm. (**b**) OSS-sEOF Buffer: as (**a**) with addition of 0.05% MHEC; conditions: as (**a**). (**c**) CSS Buffer: as (**b**); conditions: as (**a**) except 100 µA (approximately 3.50 kV). (**d**) CSS Buffer: 30 mmol/L β-alanine, 1 mmol/L poly(ethylene glycol) dicarboxylic acid, 0.1 mmol/L DETA, 0.05% MHEC, 10 mmol/L CEBCD; pH = 3.9; conditions: as (**c**) except 150 µA (approximately 3.70 kV). See Materials and Methods for all other details.

To demonstrate the capability of CSS to separate chiral compounds and to study the impact of this hydrodynamical system on chiral separations we first developed the method to separate enantiomers of PDL and PPL using the OSS-EOF by adding the negatively charged chiral selector carboxymethyl-β-cyclodextrin (CMBCD) to the separation buffer. Chiral resolution of enantiomers of PDL and PPL was achieved, as shown in Figure 2a, and resolution values are reported in Table 3. In OSS-EOF, the electroosmotic mobility (µ_EOF_) is in the direction of cathode. This was observed as a negative dip in the baseline in Figure 1a as well as in Figure 2a. The anionic cyclodextrin added to the separation buffer will exhibit under these conditions electrophoretic mobility (µ_CD_) in the direction of the anode. If |µ_EOF_| > |µ_CD_|, then the effective mobility of the chiral selector (µ_CD,eff_) will be low and equal to the difference between |µ_EOF_| and |µ_CD_|, but it will be in the same cathodic direction as EOF. If on the other hand |µ_EOF_| < |µ_CD_| then the effective mobility of the chiral selector will be in the direction of the anode. Regardless of the direction of the effective movement of the anionic chiral selector, its impact on the effective movement of cationic analytes will always be in the opposite direction of their electrophoretic mobilities, and its magnitude will be determined by the strength of the interaction between chiral selector and analytes.

Table 4 shows the relative movement and mobilities of PDL and PPL enantiomers in OSS-EOF and OSS-sEOF as well as CSS. Electrophoretic movement of positively charged ions of PDL and PPL is in the direction of the cathode and these mobilities, reported in Table 4, were obtained from experiments where the chiral selector was not present (Table 1), ignoring the impact of the ionic strength changes. The resulting effective movement of the enantiomers of PDL and PPL will be determined by the relative magnitude of the EOF, their electrophoretic mobilities and the interaction with the chiral selector. In the case of PDL, the interaction with the chiral selector is relatively weak, and the enantiomers migrate ahead of the EOF marker as it was observed in experiments where the chiral selectors were not used. However, in the case of PPL, this interaction is significantly stronger, and the enantiomers migrate after the EOF marker. The magnitude of the interaction with the chiral selector is presented in Table 4 expressed as the difference between the observed effective mobility in a separation buffer with the addition of the chiral selector and the effective mobility in a system without the chiral selector, while we assume constant electrophoretic mobilities under otherwise identical experimental conditions.

In OSS-sEOF, the observed effective mobilities of PDL enantiomers are in the direction of the cathode; however, they are significantly reduced due to the absence of EOF (Figure 2b). Moreover, the estimated interaction with the chiral descriptor is practically identical as for the OSS-EOF. Peaks for PPL enantiomers were not detected in this separation buffer. Assuming that the interaction of PPL enantiomers with the chiral selector will remain unchanged in OSS-sEOF, the effective mobilities would be in the direction of the anode and not detected on the cathodic end of the capillary as demonstrated in Table 4. Note that the numbers in italic font correspond to the calculated values which were not experimentally verified. In CSS, the electrophoretic mobilities of PDL and PPL are slightly increased probably due to elevated temperature inside the separation compartment as discussed above. However, the calculated interaction with the chiral selector was again identical to the OSS-EOF and OSS-sEOF and both enantiomers were baseline resolved (Table 3). The observed effective mobility of PDL enantiomers was, as in the OSS-sEOF, in the direction of cathode. Replacing the CMBCD chiral selector with electrophoretically less mobile carboxyethyl-β-cyclodextrin (CEBCD) resulted in a reduced interaction with PPL enantiomers leading to their baseline separation (Figure 2c,d and Table 3) and observed effective mobility in the direction of the cathode (Table 4).

### 2.3. Comparison of Hydrodynamically Opened and Closed Separation Systems for Analysis of Pharmaceuticals in Serum Samples

Blood serum represents a highly complex sample matrix, as it contains high levels of proteins as well as increased levels of salts, small molecules such as hormones and exogenous components, for example components related to pharmaceutical drugs and their metabolites. Due to this diverse composition, it represents a significant challenge for separation techniques, as it can affect their performance due to protein adsorption to the stationary phase or various components of instrumentation. In CE, adsorption of serum proteins to the capillary walls can cause problems with the reproducibility of EOF and through slow rates of association and dissociation processes between proteins and analytes it can affect peak shape and cause problems with variability of response. In extreme cases, this can result in problems with breakage of the electric current due to protein or analyte precipitation. Additionally, high salt content can cause problems with electrophoretic dispersion.

To demonstrate the suitability of CSS for complex samples we spiked PDL and PPL into a reference serum. We were unable to reliably analyze PDL and PPL spiked to untreated serum using 50 µm i.d. capillaries, as their clogging led to frequent breakages of current. In CSS carried out using wide bore capillaries we managed to perform 8–10 consecutive analyses; however, at this point, blockage of the injection device necessitated extensive cleaning. We therefore decided to reduce the amount of serum proteins through precipitation with acetonitrile followed by centrifugation. These deproteinized samples were then used to compare the performance of the three studied systems. In OSS-EOF, the first injection on a new or extensively cleaned capillary usually produced satisfactory results; however, no peaks corresponding to these compounds were observed in consecutive injections, but frequent current breakages were observed. We explained this observation by the irreversible binding of remaining serum proteins on the fused silica capillaries resulting in changes in EOF. The only way to recover these capillaries or achieve reproducible results was to carry out very extensive cleaning of the capillaries lasting up to 40 min rendering this approach impractical for routine use. This behavior was not observed for OSS-sEOF or CSS, and reproducibility of migration times, observed peak areas, obtained peak efficiencies, sensitivity and resolution for OSS-sEOF and CSS expressed as the average of ten repeated injections of the sample are listed in Table 5. From comparison of data presented in Table 1 and Table 5, it can be seen that the electrophoretic mobilities of PDL and PPL were not affected by the matrix. Additionally, results obtained for the repeatability of migration times and observed peak areas were comparable with those obtained for model samples. Marginal reduction in peak efficiency led to a slight reduction in sensitivity; however, the CSS sensitivity is approximately 40 times better than that in OSS-sEOF. Equally, sufficient resolution between PDL and PPL was observed.

**Figure 3:**
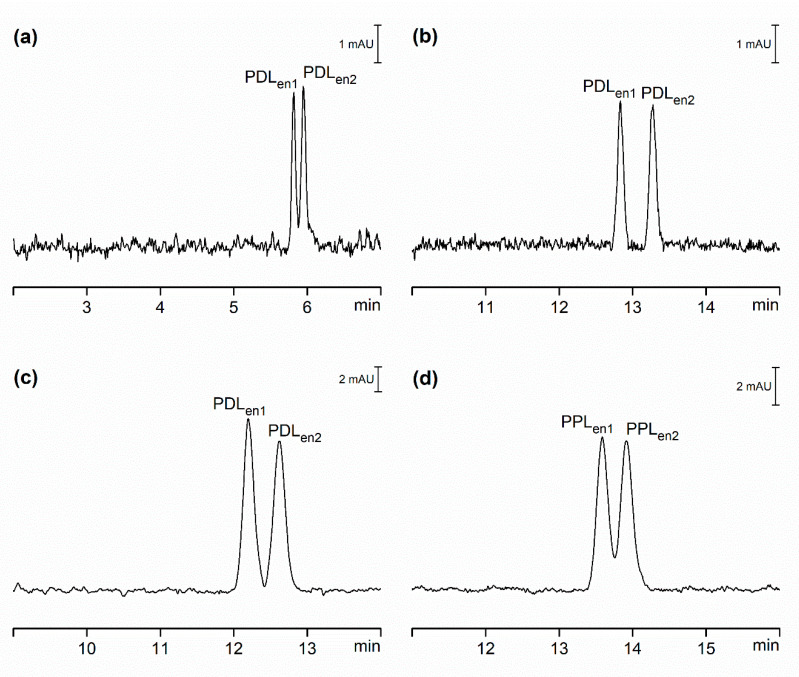
CE separation of PDL and PPL enantiomers, each prepared at concentrations of 5 µmol/L (CSS) and 20 µmol/L (OSS-EOF and OSS-sEOF) spiked into deproteinized serum. (**a**) OSS-EOF Buffer: 30 mmol/L β-alanine, 5 mmol/L poly(ethylene glycol) dicarboxylic acid, 0.1 mmol/L DETA, 2 mmol/L CMBCD; pH = 3.8; conditions: 25 kV (approximately 4.5 µA). (**b**) OSS-sEOF Buffer: as (**a**) with addition of 0.05% MHEC; conditions: as (**a**). (**c**) CSS Buffer: as (**b**); conditions: as (**a**) except 150 µA (approximately 3.90 kV). (**d**) CSS Buffer: 30 mmol/L β-alanine, 1 mmol/L poly(ethylene glycol) dicarboxylic acid, 0.1 mmol/L DETA, 0.05% MHEC, 10 mmol/L CEBCD; pH = 3.9; conditions: as (**c**) except 150 µA (approximately 3.70 kV). All other conditions as in Figure 2.

Addition of charged cyclodextrin to the separation buffer to some degree reduced the negative impact of serum protein adsorption on capillary walls as, unlike in achiral separations, we were able to observe the separation of PDL enantiomers in OSS-EOF (Figure 3a). Due to the above-mentioned reduction in EOF, PPL enantiomers were not detected on the cathodic end of the capillary. Assuming that the electrophoretic mobilities, as observed in achiral separations, are not affected by the sample matrix, then the PPL enantiomers are due a to strong interaction with CMBCD moving in the direction of anode. The same migration direction was observed for OSS-sEOF and CSS due to the absence of EOF; only PDL enantiomers were resolved and detected when the CMBCD chiral selector was employed (Figure 3b,c). Analogous to chiral separation in model samples, substitution of CMBCD for electrophoretically less mobile CEBCD led to successful separation of PPL enantiomers (Figure 3d).

When comparing chiral resolution of PDL and PPL enantiomers in model samples (Table 3) and those where analytes were spiked into the deproteinized serum (Table 6), we observed a significant increase of effective mobilities of analytes in the spiked serum samples (Table 7). This behavior was observed in both OSS-sEOF and CSS systems. Although adequate resolution of enantiomers was achieved, this was again slightly reduced in the spiked serum samples, as shown in Table 3 and Table 6. We hypothesize that this is caused by the formation of ion-pairs between residual cationic blood serum proteins and anionic cyclodextrins [35,36]. As a result, there is less cyclodextrin available for enantio-selective separation of PDL and PPL, which minimizes the reduction in µ_eff_ due to interaction with the chiral selector (Δµ_CD_) as initially described in Table 4 for model samples that do not contain serum proteins. It is worth noting that the factor by which the effective mobility is increased in spiked serum samples is nearly identical for OSS-sEOF and CSS.

## 3. Materials and Methods

### 3.1. Instrumentation and Software

The CE experiments with hydrodynamically closed separation compartment were performed using an EA Electrophoretic Analyzer (Villa-Labeco, Spišská Nová Ves, Slovakia), assembled in the single-column configuration of the separation unit equipped with a Spectra 100 photometric detector (Thermo Separation Products, San Jose, CA, USA) operating at 210 nm. Capillaries made of fluorinated ethylene-propylene copolymer (Villa-Labeco) were 30 cm (20 cm to detector) × 320 µm i.d. and 700 µm outer diameter (o.d). Samples were introduced using an injection valve equipped with a 200 nL internal sample loop (Villa-Labeco). Instrument operated at room temperature (21 °C). All data were collected and analysed using ACES (v. 1.59) software (Villa-Labeco).

The CE experiments with hydrodynamically opened separation compartment were performed using a PrinCE unit (Prince Technologies, Emmen, The Netherlands), equipped with a Lambda 1000 UV/VIS photometric detector (Bischoff Analysentechnik, Leonberg, Germany) operating at 210 nm. Fused silica capillaries (Composite Metal Services, Hallow, USA) were 70 cm (26.5 cm to detector) × 50 µm i.d. and 375 µm o.d. Samples were introduced hydrodynamically to the capillary by applying 20 mbar for 6 s. The temperature of the capillary and the samples was maintained at 21 °C. All data were collected and analyzed using Axxiom 737 v. 3.91 (Axxiom Chromatography, Moorpark, CA, USA) software.

ACD/Labs Percepta (v. 2018) was used to calculate pKa values of model analytes (ACD/pKa GALAS).

### 3.2. Chemicals and Reagents

Pharmaceutical standards of PDL and PPL (in the form of propranolol hydrochloride) were obtained from Sigma-Aldrich (Steinheim, Germany), and CMBCD and CEBCD (both in the form of sodium salt) were obtained from Cyclolab (Budapest, Hungary). Methylhydroxyethylcellulose 30,000 (MHEC; Serva, Heidelberg, Germany) was purified on a mixed-bed ion exchanger prior to addition to separation buffers at 0.05% (*w/v*) concentrations. All aqueous solutions were prepared with water demineralized by a Pro-PS purification system (Labconco, Kansas City, KS, USA). Samples containing serum were acidified by a 3 mmol/L aqueous solution of acetic acid. Acidified serum samples were either injected directly into the CE equipment or the proteins were removed by precipitation with acetonitrile before the injection. All other chemicals were of analytical grade purity.

## 4. Conclusions

Capillary electrophoresis in hydrodynamically closed separation systems facilitate the use of wide bore capillaries. Although some loss of efficiency for model samples of pharmaceutically relevant compounds was observed, this loss was proven to originate mainly from injection dispersion and only to a small part from thermal effects. While the resolution of these compounds was maintained, this loss was significantly offset by an approximately 18–23-fold increase in concentration sensitivity. The significance of this increase is particularly relevant for pharmaceutical analysis as it brings capillary electrophoresis to the required sensitivity level comparable with modern liquid chromatography. Moreover, it was demonstrated that capillary electrophoresis in hydrodynamically closed systems can be successfully applied to the chiral separation of pharmaceutical drugs in complex biological matrices such as blood serum.

## Figures and Tables

**Table 1:** Some analytical performance data for the separations of PDL and PPL in the opened (OSS-EOF and OSS-sEOF) and closed (CSS) separation systems.

Parameter	OSS-EOF		OSS-sEOF		CSS	
	PDL	PPL	PDL	PPL	PDL	PPL
µ_ep_ × 10^8^ (m^2^V^−1^s^−1^)	1.74	1.66	1.83	1.75	2.52	2.37
RSD tm (%)	0.3	0.3	0.4	0.4	0.5	0.5
RSD A (%)	2.0	1.4	3.4	2.4	2.2	1.6
N × 10^−3^ (m^−1^)	309	310	465	482	162	147
LOD (µmol/L)	1.20	1.10	1.50	1.40	0.06	0.07
Resolution ^(1)^	1.47		3.64		3.23	

µ_ep_ = electrophoretic mobility, t_m_ = migration time, A = peak area for the concentration of the analyte corresponding to 10 × LOD, N = the number of theoretical plates, LOD = concentration limit of detection. See Figure 1 for experimental conditions. ^(1)^ Resolution calculated according to [21], this applies to the entire article.

**Table 2:** Required and achieved concentration LOD for OSS-EOF, OSS-sEOF and CSS.

Required LOD				Achieved LOD (µmol/L)					
% (*w/w*)	mg/mL	µmol/L		OSS-EOF		OSS-sEOF		CSS	
		PDL	PPL	PDL	PPL	PDL	PPL	PDL	PPL
0.02	2.0 × 10^−5^	0.08	0.08	1.20	1.10	1.50	1.40	0.06	0.07

See text for assumptions made to calculate required LOD.

**Table 3:** Resolution of PDL and PPL enantiomers in studied systems.

Chiral Selector	OSS-EOF		OSS-sEOF		CSS	
	PDL	PPL	PDL	PPL	PDL	PPL
CMBCD	R_s_ = 1.33	R_s_ = 1.42	R_s_ = 5.22	r.m.	R_s_ = 2.06	n.t.
CEBCD	n.t.	n.t.	n.t.	n.t.	n.t.	R_s_ = 1.36

r.m.—reversed migration, n.t.—not tested, R_s_—resolution. See Figure 2.

**Table 4:** Observed and calculated electrophoretic and effective mobility of PDL and PPL enantiomers in studied systems.

System			PDL_en1_		PDL_en2_			PPL_en1_		PPL_en2_	
	µ_eof_	µ_ep_	µ_eff_	µ_CD_^(1)^	µ_eff_	µ_CD_	µ_ep_	µ_eff_	µ_CD_^(1)^	µ_eff_	µ_CD_
OSS-EOF	+2.20	+1.74	+2.60	−1.34	+2.56	−1.38	+1.66	+1.74	−2.12	+1.69	−2.17
OSS-sEOF	0	+1.83	+0.59	−1.24	+0.55	−1.28	+1.75	−*0.37*	−*2.12*	−*0.42*	−*2.17*
CSS	0	+2.52	+1.18	−1.34	+1.12	−1.40	+2.37	+1.08 ^(2)^	−1.29 ^(2)^	+1.05 ^(2)^	−1.32 ^(2)^

^(1)^ chiral selector CMBCD, ^(2)^ chiral selector CEBCD, µ_eof_, µ_ep_, µ_eff_—electroosmotic, electrophoretic and effective mobility respectively (×10^−8^ m^2^V^−1^s^−1^), +ve sign represents movement towards cathode, -ve sign represents movement towards anode, Δµ_CD_—reduction in µ_eff_ due to interaction with chiral selector. Values in italic font were calculated using assumptions described in text.

**Table 5:** Some analytical performance data for the separations of PDL and PPL spiked to deproteinized samples of serum in the OSS-sEOF and CSS.

Parameter	OSS-sEOF		CSS	
	PDL	PPL	PDL	PPL
µ_ep_ × 10^8^ (m^2^V^−1^s^−1^)	1.75	1.66	2.50	2.36
RSD t_m_ (%)	0.3	0.3	0.8	0.8
RSD A (%)	2.0	1.4	1.7	3.0
N × 10^−3^ (m^−1^)	392	410	151	124
LOD (µmol/L)	3.4	3.2	0.08	0.08
Resolution	3.78		2.73	

µ_ep_ = electrophoretic mobility, t_m_ = migration time, A = peak area for the concentration of the analyte corresponding to 10 × LOD, N = the number of theoretical plates, LOD = concentration limit of detection. OSS-sEOF Buffer: 30 mmol/L β-alanine, 10 mmol/L poly(ethylene glycol) dicarboxylic acid, 0.1 mmol/L DETA, 0.05% MHEC; pH = 3.7; conditions: 25 kV (approximately 3.7 µA), 21 °C, detection at 210 nm. CSS Buffer: as OSS-sEOF; conditions: as OSS-sEOF except 75 µA (approximately 2.80 kV). See Materials and Methods for all other details.

**Table 6:** Resolution of PDL and PPL enantiomers spiked into deproteinized reference serum in studied systems.

Chiral Selector	OSS-EOF		OSS-sEOF		CSS	
	PDL	PPL	PDL	PPL	PDL	PPL
CMBCD	R_s_ = 1.16	r.m.	R_s_ = 2.68	n.t.	R_s_ = 1.46	n.t.
CEBCD	n.t.	n.t.	n.t.	n.t.	n.t.	R_s_ = 1.04

r.m.—reversed migration, n.t.—not tested, R_s_—resolution.

**Table 7:** Comparison of effective mobilities of enantiomers of PDL and PPL in model samples and in spiked serum samples.

Chiral	Analyte	OSS-sEOF		CSS	
Selector		µ_eff_ × 10^8^ (m^2^V^−1^s^−1^)		µ_eff_ × 10^8^ (m^2^V^−1^s^−1^)	
		Model Sample	Spiked Serum	Model Sample	Spiked Serum
CMBCD	PDL_en1_	0.59	0.96	1.18	2.10
	PDL_en2_	0.55	0.93	1.12	2.03
CEBCD	PPL_en1_	r.m.	n.t.	1.08	1.99
	PPL_en2_	r.m.	n.t.	1.05	1.94

r.m.—reversed migration, n.t.—not tested.

## Abbreviations

CE	Capillary electrophoresis
EOF	Electroosmotic flow
HPLC	High-performance liquid chromatography
i.d.	Internal diameter
LOD	Limit of detection
CSS	Hydrodynamically closed separation system with suppressed electroosmotic flow
OSS	Hydrodynamically opened separation system
µ_ep_	Electrophoretic mobility
HDF	Hydrodynamic flow
PPL	Propranolol
PDL	Pindolol
OSS-EOF	Hydrodynamically opened separation system with electroosmotic flow
OSS-sEOF	Hydrodynamically opened separation system with suppressed electroosmotic flow
MHEC	Methylhydroxyethylcelullose
DETA	Diethylenetriamine
V_inj_	Injected volume
d	Capillary diameter
API	Active pharmaceutical ingredient
LOQ	Limit of quantitation
UHPLC	Ultra high-performance liquid chromatography
CMBCD	Carboxymethyl-β-cyclodextrin
µ_EOF_	Electroosmotic mobility
µ_CD_	Electrophoretic mobility of chiral selector
µ_CD,eff_	Effective mobility of chiral selector
CEBCD	Carboxyethyl-β-cyclodextrin
Δµ_CD_	Reduction of electrophoretic mobility due to chiral selector
o.d.	Outer diameter
